# NF-κB activity during pancreas development regulates adult β-cell mass by modulating neonatal β-cell proliferation and apoptosis

**DOI:** 10.1038/s41420-020-00386-9

**Published:** 2021-01-04

**Authors:** Dror Sever, Anat Hershko-Moshe, Rohit Srivastava, Roy Eldor, Daniel Hibsher, Hadas Keren-Shaul, Ido Amit, Federico Bertuzzi, Lars Krogvold, Knut Dahl-Jørgensen, Iddo Z. Ben-Dov, Limor Landsman, Danielle Melloul

**Affiliations:** 1 Department of Endocrinology, Laboratory of Medical Transcriptomics, Nephrology Services, Hadassah - Hebrew University Medical Center, Jerusalem, Israel; 2 Diabetes Unit, Institute of Endocrinology, Metabolism and Hypertension, Tel Aviv Sourasky Medical Center, Tel-Aviv, Israel; 3 The Sackler Faculty of Medicine Tel-Aviv University, Tel-Aviv, Israel; 4 Department of Immunology, Weizmann Institute, Rehovot, 76100 Israel; 5 Diabetes Research Institute, IRCCS San Raffaele Scientific Institute, Via Olgettina 60, 20132 Milan, Italy; 6 Paediatric Department, Oslo University Hospital HF, P. O. Box, 4950, Nydalen, 0424 Oslo Norway; 7 Laboratory of Medical Transcriptomics, Nephrology Services, Hadassah - Hebrew University Medical Center, Jerusalem, Israel; 8 Present Address: University of Copenhagen, Novo Nordisk Foundation Center for Stem Cell Biology, DanStem. Faculty for Health and Medical Sciences, Blegdamsvej 3B. DK-2200, Copenhagen, Denmark

**Keywords:** Disease model, Physiology, Cell proliferation

## Abstract

NF-κB is a well-characterized transcription factor, widely known for its roles in inflammation and immune responses, as well as in control of cell division and apoptosis. However, its function in β-cells is still being debated, as it appears to depend on the timing and kinetics of its activation. To elucidate the temporal role of NF-κB in vivo, we have generated two transgenic mouse models, the ToIβ and NOD/ToIβ mice, in which NF-κB activation is specifically and conditionally inhibited in β-cells. In this study, we present a novel function of the canonical NF-κB pathway during murine islet β-cell development. Interestingly, inhibiting the NF-κB pathway in β-cells during embryogenesis, but not after birth, in both ToIβ and NOD/ToIβ mice, increased β-cell turnover, ultimately resulting in a reduced β-cell mass. On the NOD background, this was associated with a marked increase in insulitis and diabetes incidence. While a robust nuclear immunoreactivity of the NF-κB p65-subunit was found in neonatal β-cells, significant activation was not detected in β-cells of either adult NOD/ToIβ mice or in the pancreata of recently diagnosed adult T1D patients. Moreover, in NOD/ToIβ mice, inhibiting NF-κB post-weaning had no effect on the development of diabetes or β-cell dysfunction. In conclusion, our data point to NF-κB as an important component of the physiological regulatory circuit that controls the balance of β-cell proliferation and apoptosis in the early developmental stages of insulin-producing cells, thus modulating β-cell mass and the development of diabetes in the mouse model of T1D.

## Introduction

The homeostatic control of pancreatic β-cell mass (BCM) is based on the balance of β-cell neogenesis, proliferation, and death, which are tightly controlled during development^1,2^. Islet β-cells are formed from endocrine progenitors that proliferate during the end of embryogenesis to constitute the bulk of the prenatal BCM. During the postnatal period, a significant expansion and maturation of β-cells occur to achieve an appropriate functional adult BCM^2–6^. A reduction in BCM, caused by increased apoptosis and inadequate regeneration, is a key component of type 1 (T1D) and type 2 (T2D) diabetes.

NF-κB regulates the expression of genes that play important roles in various biological processes^7,8^. NF-κB/Rel proteins exist as homo- or heterodimers, with the predominant species in islets, being the p65:50 heterodimer^9^. Numerous reports, including our own, have shown that inhibition of NF-κB in vitro prevents the acute cytokine-induced expression of deleterious genes, protecting to a large extent β-cells from apoptosis^10–17^. However, a few reports have challenged these observations by demonstrating that NF-κB stimulation protects β-cells from TNF-α-mediated apoptosis^17,18^. To clarify the in vivo role of NF-κB in β-cells, mouse models were developed where its activation is constitutively inhibited, using either the *Pdx-1/Ipf1* or the insulin promoter. While the adult transgenic mice on a wild-type background had hyperglycemia^19^, on the non-obese diabetic (NOD) background, diabetes development was accelerated^17^. More recently, Irvin et al.^20^ used a NOD transgenic mouse model expressing the NF-κB reporter luciferase chimeric gene to allow detection of activated NF-κB in the natural progression of diabetes in NOD mice. They showed that NF-κB was detectable in islets at low levels above background, but did not vary with age despite the progression of inflammatory infiltration over time.

Altogether, these reports put forward the complexity of NF-κB action, which depends on the cellular context, timing and on the kinetics of its activation^21,22^. In an attempt to control these parameters, we have generated the ToIβ-mouse model, which expresses a nondegradable IκB transgene (ΔNIκBα) in β-cells, in an inducible and controlled manner using the *tet-on* gene regulation system^14^. To further elucidate the in vivo role of the NF-κB pathway in the progression of T1D, we also generated the NOD/ToIβ mouse line^23^, which develops immune-mediated diabetes spontaneously. We, therefore, investigated whether a correlation exists between the timing of NF-κB inhibition in β-cells and the changes in disease kinetics, β-cell death, and proliferation.

In this report, we show that irrespective of the mouse genetic background, inhibiting the NF-κB pathway during the embryonic or the neonatal stage, but not during the post-weaning period, had a significant impact on BCM and β-cell turnover and on the development of diabetes on NOD background. Moreover, physiological activation of NF-κB signaling as indicated by elevated immunoreactivity of nuclear p65-subunit activation in β-cells is mainly observed at birth and during the neonatal period. However, in adult NOD mice or in pancreata from newly diagnosed patients with T1D (DiVid study^24^), low levels of cytoplasmic p65 immunostaining were detected. These findings bring the first evidence that NF-κB is involved in regulating the balance of β-cell replication and apoptosis in fetal and neonatal life, modulating β-cell turnover and therefore β-cell mass, which is determined early in development.

## Results

### Physiological expression and localization of NF-κB in insulin-expressing cells in NOD/ToIβ and ToIβ strains

**Fig. 1:**
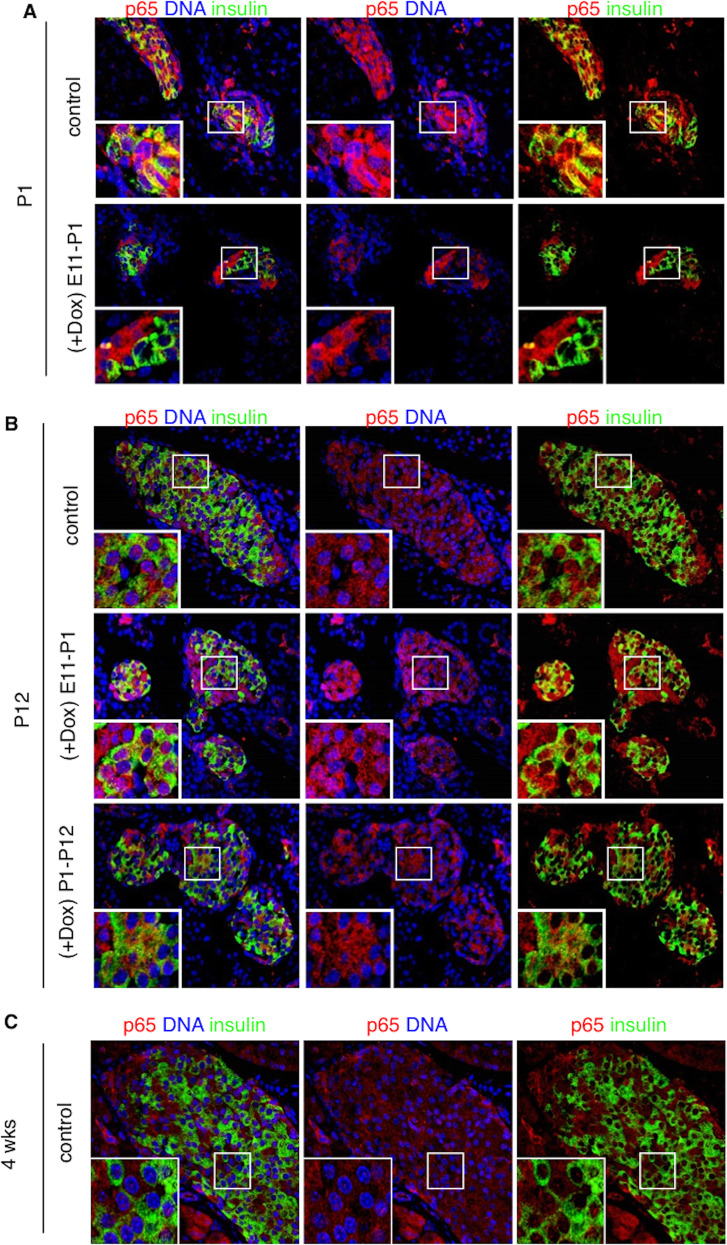
Physiological localization of the NF-κB p65-subunit in the pancreas of (A) newborn (P1), (B) neonate (P12), and (C) young adult (4 weeks) NOD/ToIβ mice. Pancreata of untreated (control) or Dox-treated during the embryonic period (E11–P1) or for 3 weeks after birth (P1–P21) were immunostained for p65 (red), insulin (green), and DNA (blue). Pictures are representative confocal images of islets from three to five different mice per group. For each mouse, ~15 islets were analyzed. Original images were taken at a magnification of ×40. Inset images were digitally increased ×4.

**Fig. 2:**
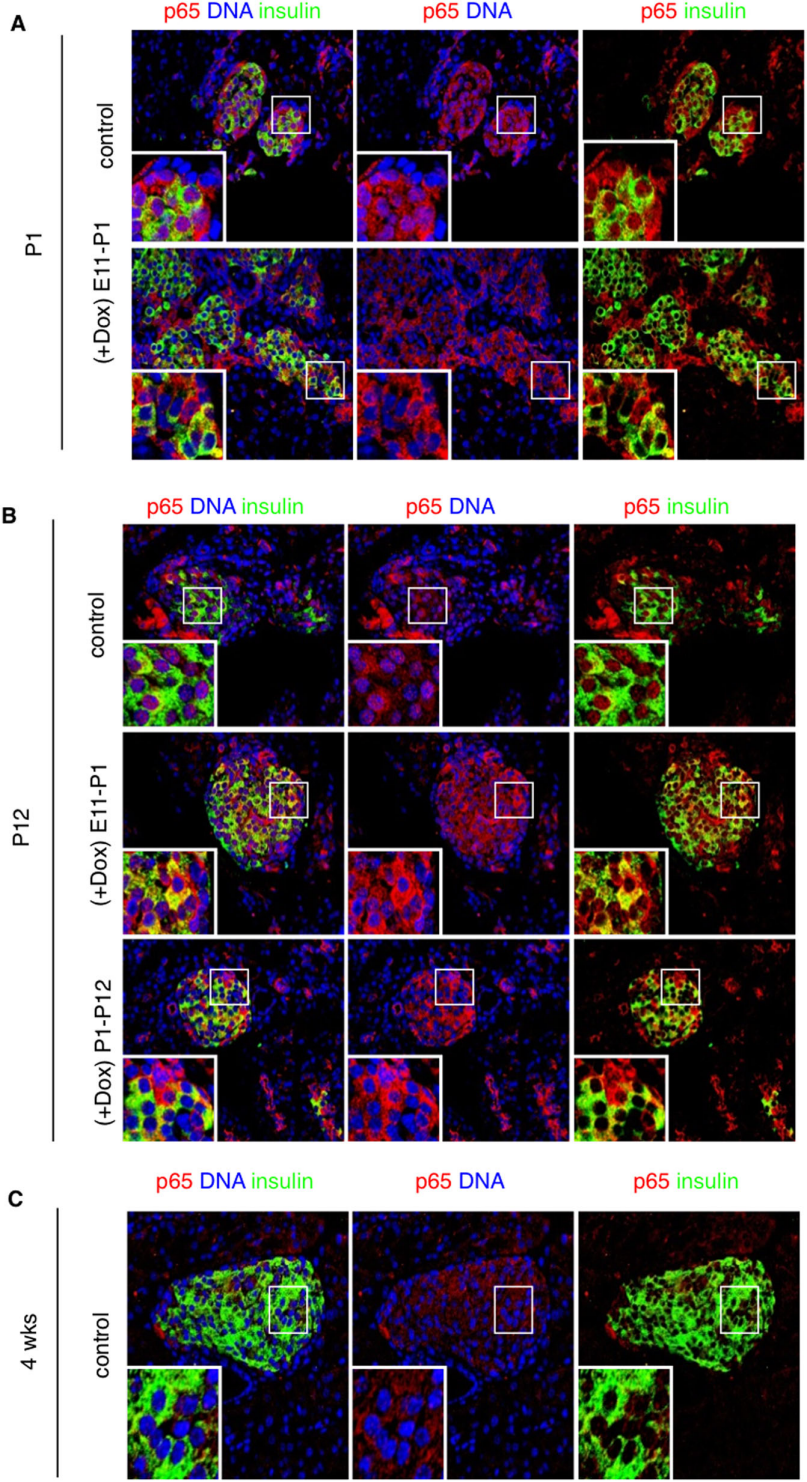
Physiological localization of the NF-κB p65-subunit in the pancreas of (A) newborn (P1), (B) neonate (P12), and (C) young adult (4 weeks) in parental ToIβ mice. Pancreata of untreated original ToIβ (control) or Dox-treated during the embryonic period (E11–P1) or for 3 weeks after birth (P1–P21) were immunostained for p65 (red), insulin (green), and DNA (blue). Pictures are representative confocal images of islets from three to five different mice per group. For each mouse, ~15–20 islets were analyzed. Original images were taken at a magnification of ×40. Inset images were digitally increased ×4.

Endocrine clusters develop relatively late in gestation and undergo substantial remodeling during the neonatal life and around the weaning period. In NOD mice, insulitis develops around 4 weeks of age with the onset of β-cell destruction occurring shortly after. We, therefore, interfered with the NF-κB pathway in β-cells, at different time periods during these developmental stages, by inducing the expression of the super-repressor ∆NIκBα, which is achieved by administration of doxycycline (Dox). Hence, we designed two sets of Dox-treated groups: in the first one, pregnant mice received Dox during the embryonic period until birth (E11–P1); in the second set, Dox was administered to nursing mothers from birth until weaning (P1–P21). Since in β-cells, the transcriptionally active form of NF-κB is mainly composed of the p65/p50 heterodimer^9,18^, we followed by immunohistochemistry, the cellular localization of the NF-κB p65-subunit in insulin-expressing cells, in newborn (P1), neonate (P12) and 4-week-old untreated (control), Dox-treated NOD/ToIβ (Fig. 1), or ToIβ (Fig. 2) mice. Surprisingly, at P1 and P12, a robust nuclear localization of the p65-subunit in insulin-expressing cells of untreated controls was detected in both mouse strains, implying a physiological activation of the NF-κB pathway (Fig. 1A, B and Fig. 2A, B; upper panels). Interestingly, from 4 weeks of age, the levels of nuclear p65 significantly dropped in insulin-positive cells (Figs. 1C and 2C). As predicted, in all the Dox-treated groups, the induced expression of the super-repressor ΔNIκBα retained the p65-subunit in the cytoplasm (Fig. 1A, B and 2A, B; lower panels). As positive controls, we used ToIβ islets incubated in vitro with TNF-α or wild-type mice injected intraperitoneally with TNF-α. Islets and liver sections were immunostained for the p65-subunit. A clear nuclear translocation of p65 in the presence of the cytokine in both islets and in liver tissue is presented (Supplementary Fig. S1a, S1b).

### Specific and temporal expression of the ΔNIκBα super-repressor in β-cells of NOD/ToIβ mice during the embryonic, neonatal, or adult period differently modulates diabetes incidence

**Fig. 3:**
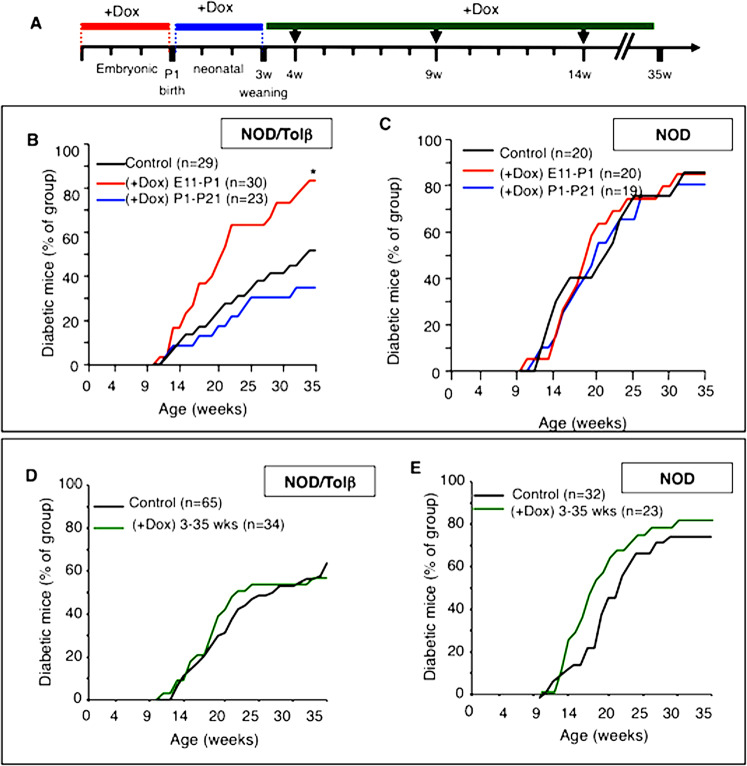
Effects of selective and conditional expression of ∆NIκBα in insulin-producing cells during embryonic, neonatal, or adult periods on diabetes incidence in NOD/ToIβ as compared to the parental NOD/LtJ female mice. **A** Experimental design: NOD/ToIβ or NOD/LtJ female mice were exposed to Dox (0.2 mg/ml) during the embryonic period until birth (E11–P1: red line), during the neonatal period from birth to weaning (P1–P21: blue line) or after weaning (3–35 weeks) up to 35 weeks (green line). Diabetes incidence was monitored once a week until 35 weeks. **B** Percentage of hyperglycemic NOD/ToIβ mice in untreated (control, black line, *n* = 29) or Dox-treated animals during the embryonic period until birth (red line, *n* = 30); from birth until weaning (blue line *n* = 23) or **D** between 3 and 35 weeks (green line, *n* = 34). *P < 0.008 vs control. **C**, **E** Percentage of hyperglycemic mice in the parental NOD/LtJ female mice similarly treated as in **B** and **D**. **C** NOD/LtJ mice untreated (control, black lane *n* = 20); or Dox-treated animals during the embryonic period until birth (red line, *n* = 20); from birth until weaning (blue line *n* = 19). **E** Diabetes incidence in untreated (control; black line, *n* = 65) and Dox-treated animals from 3 weeks onward until the end of the follow-up at 35 weeks (green line, *n* = 23).

We next examined whether a possible association exists between the NF-κB pathway in β-cells and the development of diabetes in NOD/ToIβ mice. Cumulative evidence unveiled the existence of a checkpoint at 3 weeks of age in the development of diabetes in NOD^25^. We therefore set, in addition to the two described Dox-treated groups during embryonic (E11–P1) or neonatal (P1–P21) periods, a third group where Dox was administered immediately after weaning until the end of the follow-up at 35 weeks of age (3–35 weeks) (Fig. 3A). The untreated NOD/ToIβ mice served as controls. Interestingly, adult NOD/ToIβ mice in which ΔNIκBα was induced in insulin-expressing cells during the embryonic period (E11–P1) had a significantly higher incidence of diabetes at 35 weeks (83%) compared to the control group (52%), (Fig. 3B). Of note, in all groups of mice in which ΔNIκBα was induced during the embryonic period either until weaning (E11–P21) or throughout the adult life (E11–35 weeks), the diabetes incidence was also significantly higher (Supplementary Fig. S3). Inversely, induction of the super-repressor during the neonatal period (P1–P21) showed a trend towards decreased diabetes incidence (35%, Fig. 3B). However, when the activation of the NF-κB pathway was inhibited from weaning onward, the diabetes incidence was similar in Dox-treated and in untreated NOD/ToIβ mice (Fig. 3D, 3–35 weeks vs control).

It was suggested that hyperglycemia prevalence in NOD mice is influenced by changes in the microbiome^26^, which in turn can affect the immune system. We, therefore, carried out the controlled experiment of testing whether Dox by itself had any effect on diabetes incidence. We repeated the same sets of experiments as designed in Fig. 3A, using the parental NOD/LtJ mice. Figures 3C, E show no effect of the antibiotic on the development of diabetes.

We next tested the hypothesis of whether the increased diabetes incidence obtained in the fetal E11–P1 group of NOD/ToIβ mice predisposed the progeny to a higher risk of developing diabetes. For that, we generated second-generation offspring by mating F1 NOD/ToIβ mice born from Dox-treated parents and monitored the blood glucose levels of F2 females. Similar to the results presented in Fig. 3B, a high incidence of diabetes was observed in F1 as compared to untreated mice. However, in the F2 generation NOD/ToIβ mice, the incidence was similar to that in control mice (Supplementary Fig. S2),

Our data indicate that preventing NF-κB activation in insulin-expressing cells during the fetal period has a significant long-term effect on diabetes incidence that is not transmitted to the next generation. In contrast and in correlation with the lack of NF-κB nuclear localization in older mice, blocking its activation in β-cells throughout adulthood has no impact on the development of the disease.

### Lack of an apparent role for NF-κB in adult NOD/ToIβ

**Fig. 4:**
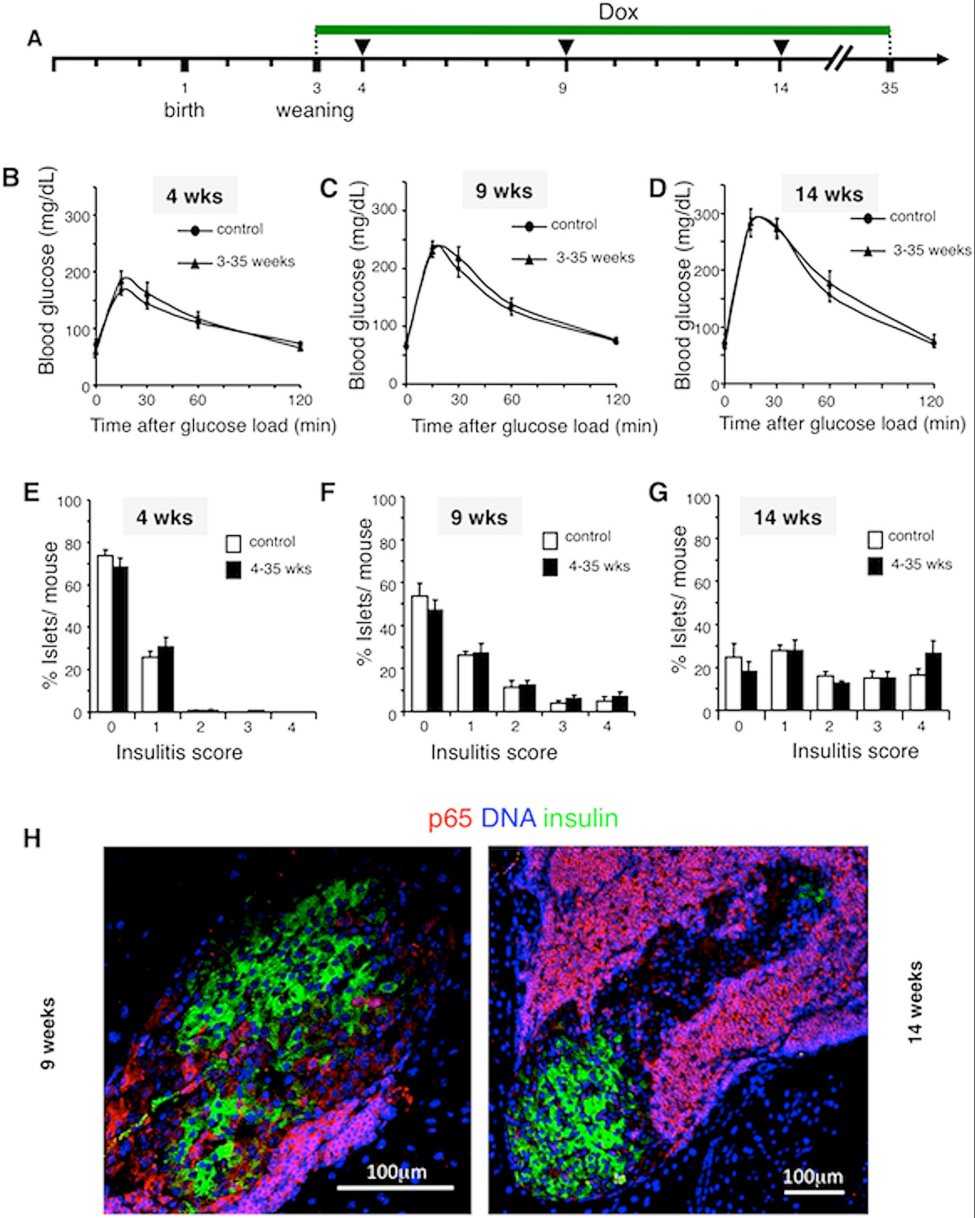
Selective and conditional inhibition of the NF-kB pathway in β-cells during adulthood has no effect on glucose tolerance or insulitis in the transgenic NOD/ToIβ mice. **A** Experimental design: NOD/ToIβ female mice were exposed to Dox from weaning to 35 weeks. **B**–**D** IPGTT was performed at 4 (**B**), 9 (**C**) or 14 (**D**) weeks of age. The results are presented as the mean ± SEM of data pooled from untreated controls (circles, *n* = 10–16), Dox-treated 3–35 weeks (triangles, *n* = 8–11). **E**–**G** Insulitis was assessed using hematoxylin and eosin staining of histological sections of the pancreata of untreated (empty bars) or Dox-treated (black bars) mice. Insulitis score was calculated as a percent of total islets per mouse at (**E**) 4 weeks: control, (487 islets, *n* = 5) and 3–35 weeks (329 islets, *n* = 4); at (**F**) 9 weeks: control (249 islets, *n* = 4) and 3–35 weeks (355 islets, *n* = 4); and at (**G**) 14 weeks: control (336 islets, *n* = 5) and 3–35 weeks (361 islets, *n* = 5). Values are mean ± SE. **H** Pictures are representative confocal images of islets from three to four different mice per group taken at 9 weeks of age. For each mouse, ~20 islets were analyzed. Pancreata were immunostained for p65 (red), insulin (green), and DNA (blue). Original images were taken at a magnification of ×40.

Results presented in Fig. 3D demonstrated that expression of ∆NIκBα from weaning up to 35 weeks led to a similar diabetes incidence between Dox-treated and untreated NOD/ToIβ mice. Moreover, no activated nuclear NF-κB p65-subunit was detected in islets of 9- or 14-week-old animals despite significant peri- and intra-islet insulitis (Fig. 4H). To determine whether ∆NIκBα expression had any impact on β-cell function under stress conditions, we performed IPGTT and tested the glucose clearance at 4, 9, and 14 weeks of age. Among all age groups, similar blood glucose levels were measured in untreated and Dox-treated groups (Fig. 4B–D). The progression of the immune reaction as measured by the different degrees of insulitis also showed no differences between treated and untreated groups (Fig. 4E–G).

These results indicate that inhibiting the NF-κB pathway in β-cells post-weaning does not contribute to immune-mediated diabetes in the NOD model of T1D.

### Embryonic expression of ∆NIκBα super-repressor in insulin-expressing cells exacerbates glucose tolerance along with higher insulitis severity in adult NOD/ToIβ mice

**Fig. 5:**
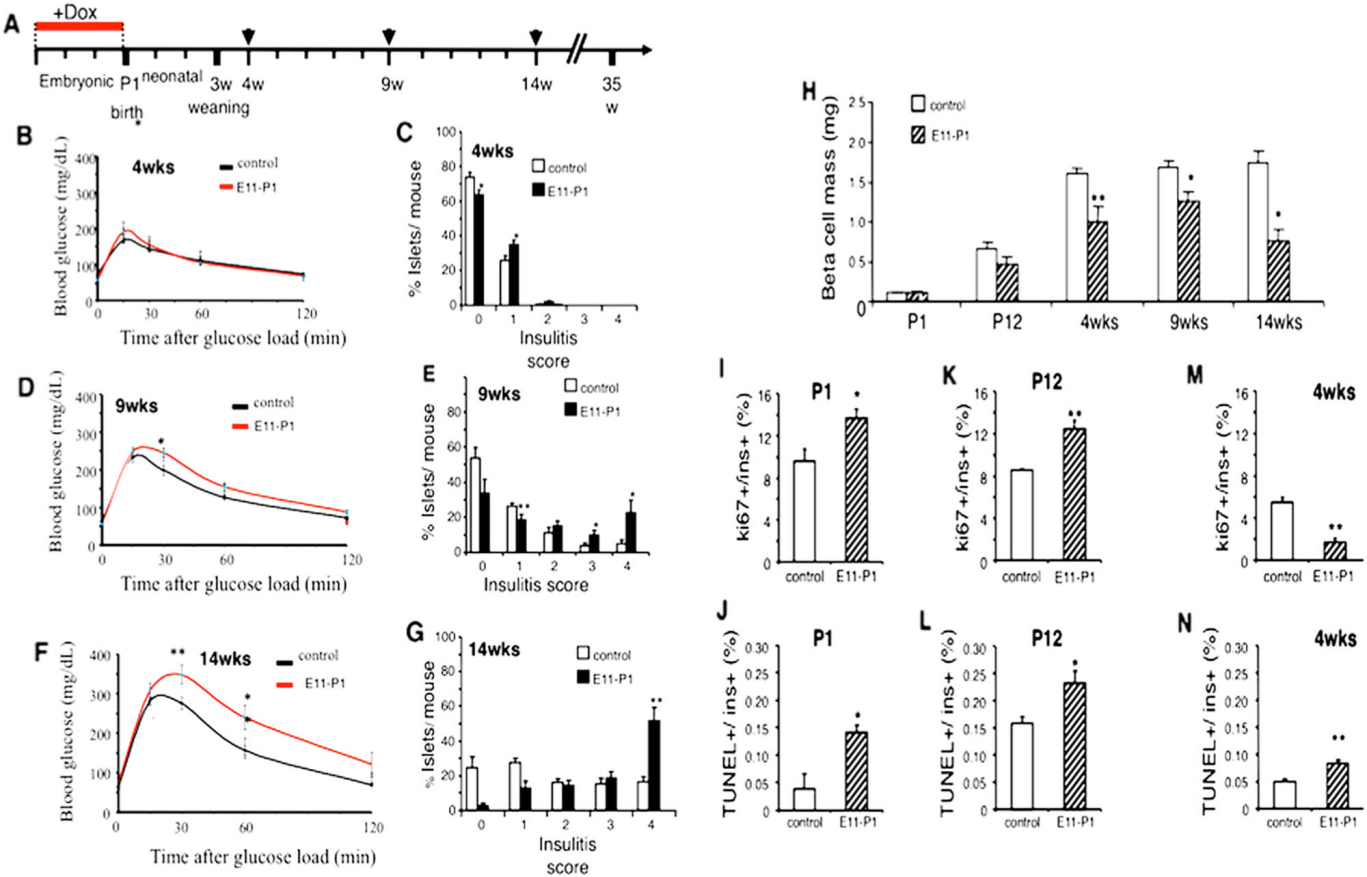
Effect of selective and conditional expression of ∆NIκBα in insulin-producing cells during the embryonic period on glucose tolerance, the degree of insulitis, β-cell mass (BCM), β-cell proliferation, and apoptosis in NOD/ToIβ mice. **A** Experimental design: NOD/ToIβ or NOD/LtJ female mice were exposed to Dox during the fetal period until birth (E11–P1). IPGTT was performed at 4 (**B**), 9 (**D**), or 14 **(F**) weeks of age. After an overnight fast, mice were injected with 2 g/kg D-glucose and blood samples were collected at 0, 15, 30, 60, and 120 mins post-loading, and glucose levels were measured. The results are presented as the mean ± SEM of data pooled from untreated controls (black lines, *n* = 10–16), Dox-treated E11–P1 (red lines, *n* = 12–13). *P < 0.05 vs control. **C**, **E**, **G** Insulitis scores calculated for 4, 9, and 14 weeks of age: **C** control, (487 islets, *n* = 5), E11–P1 (436 islets, *n* = 5) at 4 weeks; at 9 weeks (**E**): control (249 islets, *n* = 4), E11–P1 (457 islets, *n* = 5); and at 14 weeks (**G**): control (336 islets, *n* = 5), E11–P1 (169 islets, *n* = 6). Values are mean ± SEM. *P < 0.04, **P < 0.009 vs control. **H** BCM was determined and is presented in milligrams: mice were sacrificed on P1: *n* = 3; P12: *n* = 4; 4 weeks: *n* = 3–5; 9 weeks: *n* = 3–5 and 14 weeks: *n* = 3–4): controls (empty bars) or Dox-treated (hatched bars). Values are mean ± SEM. *P < 0.05, **P < 0.01 vs control. **I**, **K**, **M** β-cell proliferation was assessed by co-staining for Ki67, insulin, and calculated as a percent of Ki67-positive nuclei in insulin-positive cells out of the total number of insulin-positive cells per mouse. **I** P1 (*n* = 4–5, ~4550 β-cells); **K** P12 (*n* = 4, ~7550 β-cells); **M** 4 weeks (*n* = 3–4, ~8400 β-cells). **J**, **L**, **N** β-cell apoptosis was assessed using TUNEL assay and co-staining for insulin and calculated as a percent of TUNEL-positive nuclei in insulin-positive cells out of the total number of insulin-positive cells per mouse. **J** P1 (*n* = 4–5, ~4600 β-cells); **L** P12 (*n* = 4, ~9750 β-cells); **N** 4 weeks (*n* = 3–4, ~16,400 β-cells). *P < 0.05.

Since ∆NIκBα expression during the fetal period led to increased diabetes incidence in adult mice, we sought to elucidate the long-term effects of the super-repressor on pancreatic β-cell function, and the severity of the autoimmune process. We, therefore, performed IPGTT and assessed the degrees of insulitis on normoglycemic 4-, 9-, and 14-week-old mice in untreated (control) or Dox-treated (E11–P1) NOD/ToIβ mice. The degree of insulitis was calculated as the percent of islets with a specific score (0–4), as previously described^14^. As expected, with age, both groups showed a progressive impairment of blood glucose clearance accompanied by increased severity of insulitis. However, in the E11–P1 group, a slight decrease in blood glucose clearance was already detected at 4 weeks of age 15 min after the glucose load (Fig. 5B) as well as a small, yet statistically significant change in the insulitis score distribution (Fig. 5C). With time, there was an additional deterioration in blood glucose clearance (Fig. 5D), accompanied by a further increase in islets displaying a higher insulitis score (Fig. 5E). At 14 weeks of age, more pronounced differences were observed, as mice showed overt glucose intolerance compared to the untreated NOD/ToIβ (Fig. 5F), accompanied by a marked increase in immune infiltration (Fig. 5G).

### Embryonic expression of ∆NIκBα in insulin-expressing cells alters β-cell mass and β-cell turnover in both NOD/ToIβ and ToIβ

**Fig. 6:**
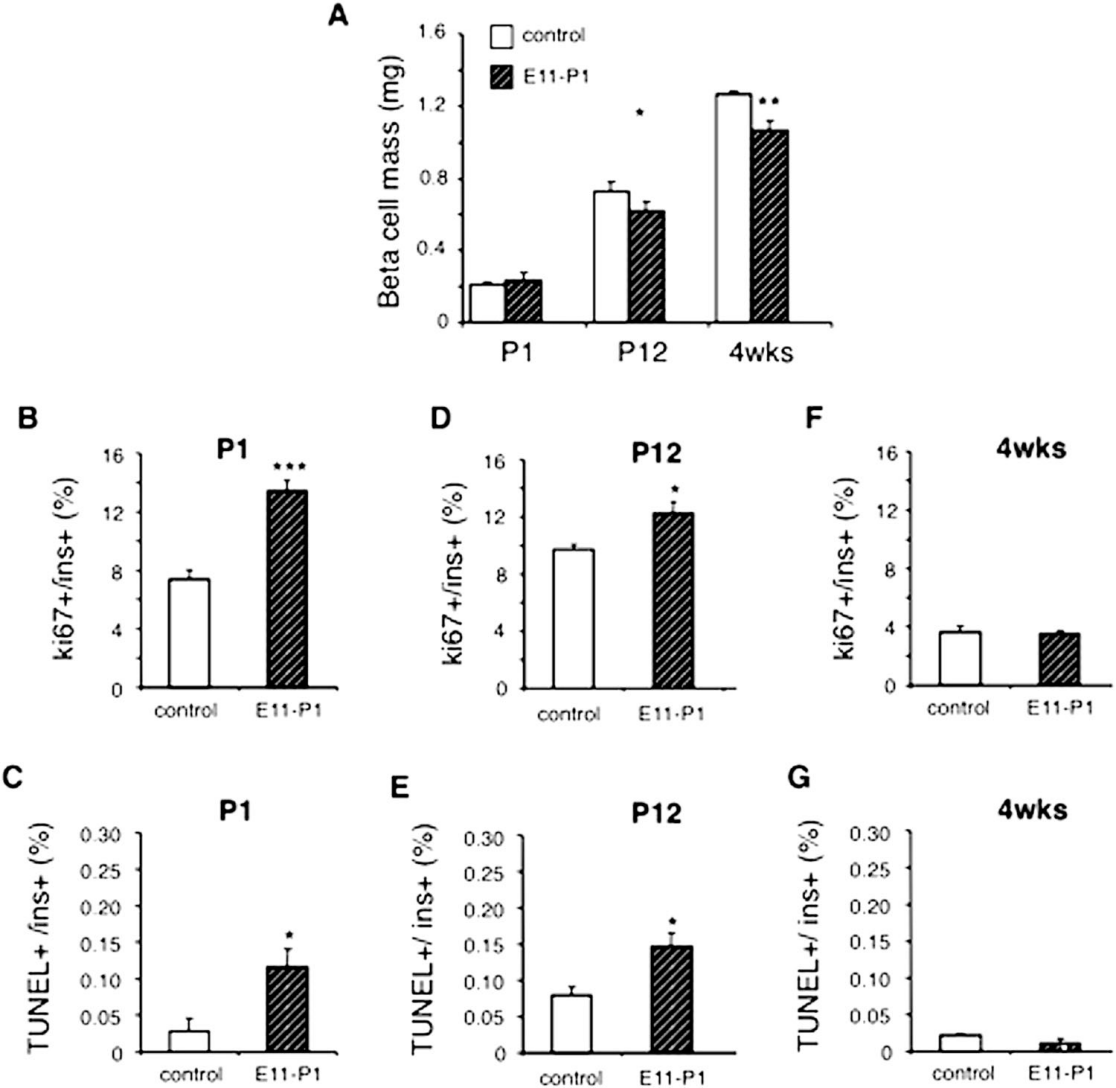
Effect of ∆NIκBα induced expression in insulin-producing cells during the embryonic period on β-cell mass, β-cell proliferation, and apoptosis in non-diabetic transgenic ToIβ mice. **A** BCM was determined by immunostaining for insulin and is presented in milligrams. **B**, **D**, **F** β-cell proliferation was assessed by co-staining for Ki67, insulin, and calculated as a percent of Ki67-positive nuclei in insulin-positive cells out of the total number of insulin-positive cells per mouse. **B** P1 (*n* = 5, ~6100 β-cells); **D** P12, (*n* = 4, ~8800 β-cells); **F** 4 weeks (*n* = 3–4, ~11,150 β-cells). **C**, **E**, **G** β-cell apoptosis was assessed using TUNEL assay and co-staining for insulin and calculated as a percent of TUNEL-positive nuclei in insulin-positive cells out of the total number of insulin-positive cells per mouse. **C** P1 (*n* = 5–6, ~5160 β-cells; **E** P12 (*n* = 4, ~13,250 β-cells); **G** 4 weeks (*n* = 3–4, ~19,000 cells). Values are mean ± SEM. *P < 0.05, **P < 0.009, ***P < 0.0004 vs control.

Next, we measured the BCM in the pancreata isolated from untreated and Dox-treated E11–P1 groups in both the NOD/ToIβ (Fig. 5H) and the non-diabetic ToIβ (Fig. 6A) strains. BCM in untreated NOD/ToIβ exhibited a linear increase, with a sharp slope from P1 to 4-week-old animals, reaching a plateau between 4 and 14 weeks of age (Fig. 5H, control, empty bars). The E11–P1 Dox-treated group exhibited a significant reduction in BCM (Fig. 5H, hatched bars). The drop in BCM older mice correlated with the increased severity of insulitis and acceleration of the immune reaction (Fig. 5E–G).

To assess whether the effect of NF-κB pathway inhibition on BCM was specific to the autoimmune background, we used the founder ToIβ mice in which ∆NIκBα was similarly and temporally expressed during the fetal period. Interestingly, a similar picture emerged at 4 weeks of age, where BCM decreased in Dox-treated animals (E11–P1) (Fig. 6A, hatched vs empty bars).

To elucidate the drop in BCM, we measured β-cell proliferation and apoptosis in the newborn (P1), neonate (P12), and young adult (4 weeks) of both transgenic lines. Surprisingly, in the E11–P1 NOD/ToIβ group, the levels of both β-cell proliferation (Fig. 5E: 142% of control) and apoptosis (Fig. 5J: 365% of control) were elevated already at P1. Remarkably, these parameters continued to be elevated at P12, days after the induction of the transgene was terminated (Fig. 5K: 145% of control and Fig. 5L: 147% of control). At 4 weeks, however, the NOD/ToIβ showed a dramatic drop in proliferation, whereas apoptosis was still elevated (Fig. 5M: 30% of control; Fig. 5N: 169% of control), resulting in the net decrease in BCM shown in Fig. 5H.

In the ToIβ transgenic line, the temporal inhibition of NF-κB presented an overall similar picture on days P1 and P12. Embryonic ∆NIκBα expression (Fig. 6, hatched bars) led to increased proliferation and apoptosis at both P1 (Fig. 6B, C: 182 and 404% of control) and P12 (Fig. 6D, E: 126 and 185% of control,). However, unlike the autoimmune-prone model, at 4 weeks of age, the levels of both proliferation and apoptosis dropped significantly and were similar in both untreated and Dox-treated ToIβ mice (Fig. 6F, G). We assume that the apoptotic cells being cleared by resident phagocytes, the number of TUNEL-positive cells presented in Figs. 5 and 6 could be underestimated and the ratio of apoptosis to proliferation levels may explain the net decrease in BCM (Figs. 5H and 6A, hatched bar).

Taken together, the data suggest that the selective and temporal blockade of the NF-κB activation during embryogenesis has led to an increase in β-cell turnover and ultimately to a decrease in BCM long after the termination of ΔNIκBα-induction independently of the genetic background of the mice.

### Gene expression analysis in islets of mouse neonates following embryonic expression of ∆NIκBα in insulin-expressing cells in NOD/ToIβ mice

**Fig. 7:**
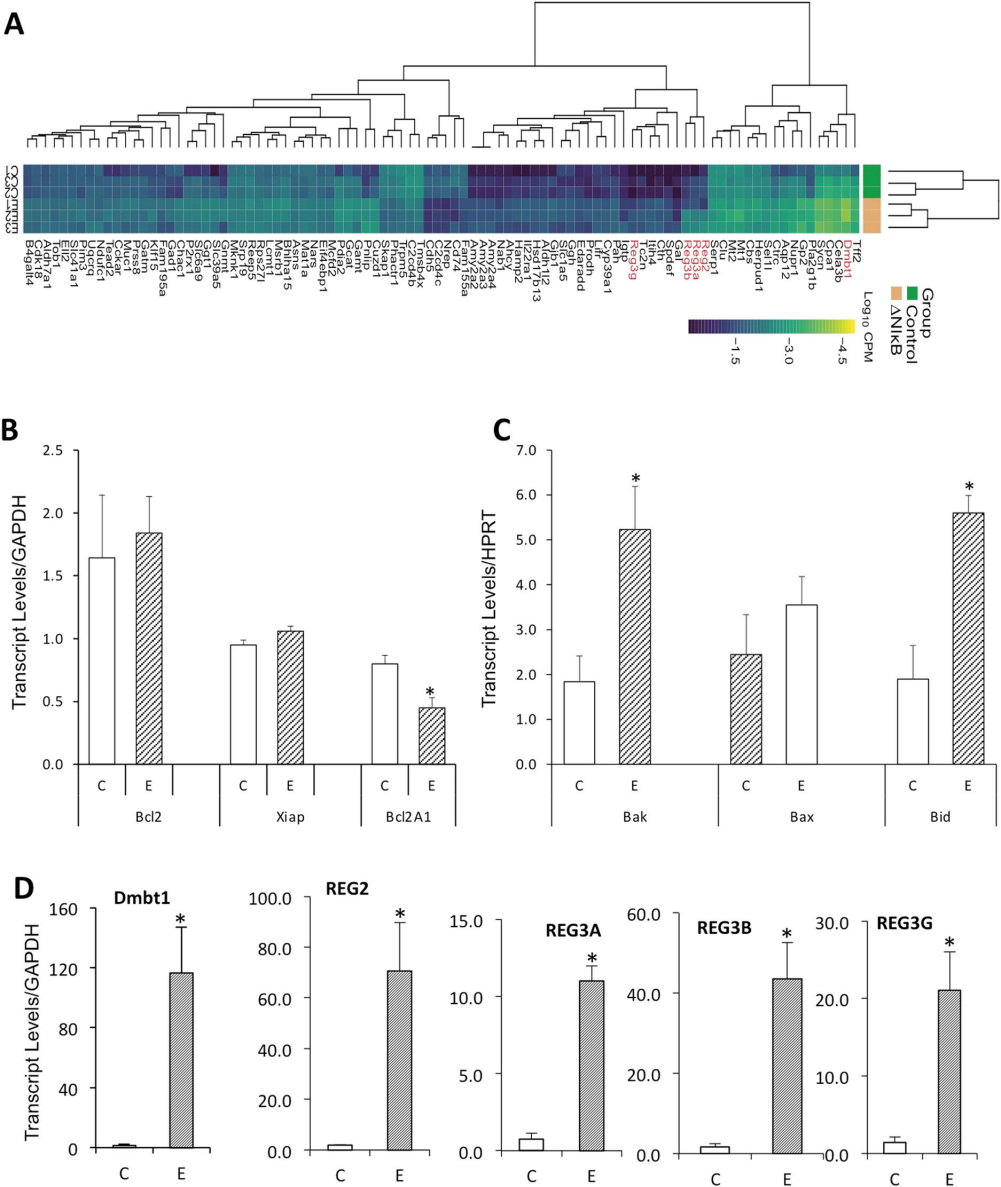
Embryonic inhibition of the NF-κB pathway in insulin-expressing cells differentially regulates the expression of genes in islets of young mice as evaluated by RNA-Seq. **A** Heatmap showing differentially expressed genes in islets isolated from 3-week-old mice where ∆NIκBα was transiently expressed during the embryonic period (E1, E2, E3) as compared to controls (C1, C2, C3). mRNAs were hierarchically clustered using Ward’s method and Manhattan distance metric. In the heatmap, darker shades represent a higher expression. *Reg* family genes and *Dmbt1* are depicted in red. **B** (anti-apoptotic genes), **C** (pro-apoptotic genes), **D** (genes associated with regeneration). Validation by RT-qPCR panel of mRNA levels of selected genes in control islets (C-Empty bars) and in islet isolated from NOD/ToIβ in which NF-κB pathway was inhibited during the embryonic period (E-hatched bars). *GAPDH or HPRT* served as an internal standard. The data are presented as the mean ± SEM of *n* = 3–4 experiments. *P values < 0.05.

Islets undergo substantial remodeling during neonatal life. We first investigated the expression of pro- and anti-apoptotic genes in islets from 3-week-old NOD/ToIβ mice of untreated control (C) or Dox-treated during the embryonic period (E) that could explain the reduction in BCM. While the prosurvival Bcl-2-homolog BCL2A1 mRNA levels, reported to be a direct transcriptional target of NF-κB^27^ were significantly reduced (Fig. 7B), a major increase in the expression of the pro-apoptotic BH3-only Bid and the effector Bak genes was observed (Fig. 7C). However, no difference in Bcl-2^28^, Bax^29^, or XIAP gene expression was obtained (Fig. 7B). The latter was suggested to play an important role in β-cell apoptosis and to be involved in the pathogenesis of T1D^30^.

To identify additional differentially expressed genes or molecular pathways during the neonatal period, RNA-seq profiling was performed using pooled islets from 2 to 3 weeks-old NOD/ToIβ mice, in which ΔNIκBα was induced during the embryonic period (E1–E3), compared to controls (C1–C3). Heatmap showing differentially expressed genes using Ward’s method and Manhattan distance metric is presented in Fig. 7A. The list of differentially expressed genes, based on their false detection rate (FDR) < 0.1 and a *P* value < 0.05, is presented in Supplementary Table S1. Interestingly, this analysis revealed a significant upregulation of members of the regenerating (REG) family of genes. Validation by real-time PCR of these genes was performed and is presented in Fig. 7D, including REG2 (70.5 ± 19 vs 1.9 ± 0.2, *P* value = 0.028), REG3A (11.6 ± 2.5 vs 0.74 ± 0.14, *P* value = 0.0173); REG3B (43.5 ± 9 vs 1.71 ± 0.75, *P* value = 0.01) and REG3G (21 ± 4.9 vs 1.42 ± 0.7, *P* value = 0.012), DMBT1 (deleted in malignant brain tumor 1) (116.4 ± 30.5 vs 1.5 ± 0.9). Of note, DMBT1 was previously suggested to be a putative marker of islet precursor cells^31^.

### Lack of detectable levels of activated nuclear NF-κB p65-subunit in insulin-expressing cells of recently diagnosed patients with T1D

**Fig. 8:**
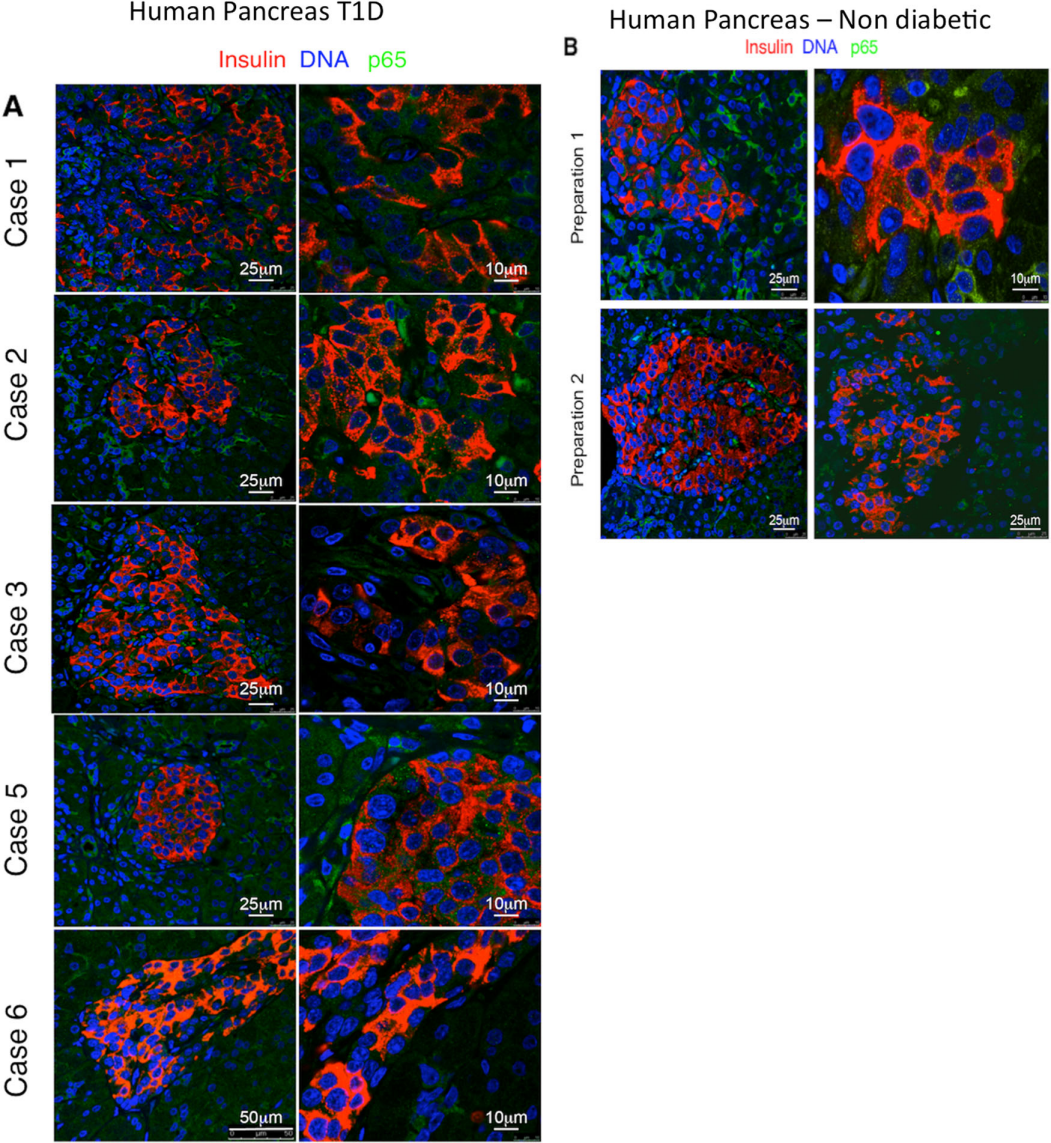
Immunolocalization of the NF-κB p65-subunit in the pancreas of recently diagnosed human T1 patients. **A** Laparoscopic pancreatic tail resection samples of five T1D patients (Divid-Study Patient cases 1–3, 5, 6) and **B** of two representative non-diabetic pancreata were immunostained for p65 (green), insulin (red), and DNA (blue). Pictures are representative confocal images of islets from 9 to 27 islets per group. Original images were taken at a magnification of ×25 left panel and ×10 right panel.

We finally tested whether the lack of nuclear localization of the p65-subunit in insulin-positive cells in NOD mice was specific to the mouse model of T1D, or was this observation also applies to β-cells of T1D patients. Hence, we examined the localization of the p65 by immunohistochemical staining in laparoscopic pancreatic tail resection samples obtained from five recently diagnosed T1D patients (median 5 weeks, as described in the DiViD study^24^) (Fig. 8A) and in the pancreas from non-diabetic individuals (Fig. 8B). Tissue sections were analyzed using antibodies against p65 and insulin. Similar to the results obtained in NOD/ToIβ, no nuclear translocation was detected in the pancreas of any of the 5 T1D cases despite the presence of insulitis nor in that of the two non-diabetic individuals (Fig. 8A, B).

These findings support the data presented above using the NOD model of T1D regarding the lack of a clear association between the activation of the NF-κB pathway and the manifestation of diabetes in mice or in recently diagnosed patients.

## Discussion

During physiological growth in neonates, β-cells adapt to their environment by enhancing their function and growth to achieve appropriate functional BCM. The role of NF-κB in β-cells has been the focus of several studies with different results and conclusions, leading to either protective or destructive effects, depending on the experimental settings used. The mouse models presently used enabled us to assess the physiological role of NF-κB in insulin-expressing cells and evaluate the effect of its controlled inhibition during specific time intervals.

In this report, we present evidence that on the NOD background, blocking the activation of the NF-κB pathway from weaning and throughout adulthood had no effect on diabetes incidence, glucose tolerance and the degree of insulitis. Remarkably, in all NOD/ToIβ groups in which ΔNIκBα was expressed during the embryonic period (E11) until birth (E11–P1), until weaning (E11–P21), or throughout the adult life (E11–35 weeks), the diabetes incidence was significantly higher compared to the untreated group.

The lack of apparent roles of the NF-κB pathway in adult mice correlates with the weak immunoreactivity of p65-subunit detected in β-cells in both the NOD/ToIβ and ToIβ mice after weaning, with levels remaining low even in the presence of severe insulitis in NOD/ToIβ. This finding agrees with the reported background levels of luciferase-reporter activity under the control of NF-κB elements in NOD that did not vary with age, despite the inflammatory infiltration in islets^20^. Importantly, in pancreatic resection biopsies from recently diagnosed T1D individuals^24^, no clear nuclear translocation of NF-κB p65-subunit was detected. This observation supports the findings of low or undetectable expression levels of chemokines and cytokines known to be NF-κB target genes, in islets from these patients^32^.

In rodents, it appears that the fetal life represents a defined time-window in which the appropriate number of β-cells and that of progenitor cells are established defining BCM during organ development^6^. Disturbance of this process is exemplified in models of intrauterine growth restriction, in which the number of β-cells may initially be adequate. Later however, the BCM becomes unable to compensate for the increased body needs. The hypothesis that an abnormal intrauterine milieu such as maternal obesity can induce permanent changes in glucose homeostasis after birth and lead to diabetes in adulthood has been shown in the NOD model^33^ and was also suggested to be associated with an increased incidence of T1D in children^34^. It is conceivable that environmental triggers could affect the NF-κB pathway, therefore influencing the development of T1D in predisposed subjects. Importantly, using the current T1D model, the changes that occurred in the F1 generation of the fetal E11–P1 group and which led to the high diabetes prevalence were not transmitted to the next generation.

The balance between β-cell apoptosis and replication determines the postnatal regulation of BCM. We, therefore, measured BCM at different ages and found that mice with the inhibited NF-κB pathway during the embryonic period show a trend towards reduced BCM in neonates as early as 12 days and become more pronounced with age. The clear reduction in BCM in the non-diabetes ToIβ mice is of the utmost importance since it reveals for the first time the existence of a physiological role for NF-κB during pancreas development. It is interesting to note that in control mice, an increase in β-cell apoptosis on day 12 (P12), followed by a drop at 4 weeks back to the levels measured at birth. This is in accordance with reported studies showing that remodeling of the neonatal pancreas involves a wave of β-cell apoptosis peaking at around P12 in the mouse^35–38^. In fact, it was hypothesized that this wave of β-cell apoptosis could play a role in triggering β-cell-directed autoimmunity^38,39^.

A set of genes playing a role in apoptosis that could explain the reduction of the β-cell number in the E11–P1 group was initially identified. A significant decrease in the prosurvival Bcl-2-homolog BCL2A1/Bfl-1 gene expression was observed. This gene was previously reported to be a direct target of NF-κB and its expression was also inhibited by a similar IκB super-repressor^40^. A shared characteristic of all anti-apoptotic Bcl-2 proteins is the sequestration of pro-apoptotic proteins, including the multidomain proteins Bak/Bax. In this regard, Bcl2A1was reported to have a robust association with the endogenous Bak but not with Bax^27,41^. Interestingly, we found a strong upregulation of Bak and Bid gene expression in islets isolated from the E11–P1 group. The binding of Bid to Bak triggers the unfolding event that leads to the formation of homodimers and subsequently to the generation of pores on the mitochondrial outer membrane via homo-oligomerization. Cytochrome *c* and other apoptotic factors are then released initiating downstream apoptosis events culminating in the cell’s demise^42^.

Remarkably, gene expression profiling in islets from the E11–P1 group revealed a number of genes that belong to the REG (regenerating) family. REG proteins constitute a conserved family of C-type lectin proteins, which have been implicated in regenerating islets. REG protein family upregulated expression was suggested to reflect the effort of the islets to compensate for the loss of β-cells. Despite this, in T1D, BCM continues to drop because the endogenous regenerative response is overwhelmed by the ongoing autoimmunity. REG proteins may also act as autoantigens contributing to activate lymphocytes, off-balancing the loss and generation of β-cells^43^. One of the highly upregulated genes is Reg2. An increased Reg2 level has been reported in a newly diagnosed T1D patient and was also shown to act as T-cell autoantigen in NOD mice^44^. In addition, the expression of other members of the *Reg3* family of genes, which includes Reg3A, Reg3G, and Reg3B was enhanced. Thus, REG proteins that are increased in our model could play a dual role as β-cell trophic factors and autoantigens at different stages of T1D, but their precise roles remain to be clarified in the context of NF-κB signaling perturbation.

DMBT1 was remarkably upregulated in islets of the E11–P1 group. Interestingly, using an in vitro generation system of islets from human pancreatic tissue, DMBT1 was differentially expressed at a stage that has a large population of precursor cells, during the progression to differentiated cells, making it a candidate marker of precursor cells or cells in the early stages of endocrine differentiation^31^. Overall, these reports support our observation that β-apoptosis is associated with the upregulation of genes linked to β-cell proliferation and differentiation in attempts to counteract the destruction of β-cells, resulting in the increased β-cell turnover.

The selective blockade of NF-κB activation in a temporal manner had a long-term impact on β-cell function and turnover, days to weeks after the restoration of the NF-κB pathway to normal. This points towards a potential role for epigenetic changes in β-cells that need to be further identified. Actually, the role of epigenetic modifications in NOD mice has been suggested in a study where I-BET151, an inhibitor of bromodomain-containing transcriptional regulators, inhibited the development of diabetes^45^. The direct binding of the Brd4 to the p65/RelA has been documented^46^. In agreement with our results where NF-κB was solely inhibited in β-cells, the subset of genes whose expression was increased most by the inhibitor in these cells also encoded multiple members of the REG protein family^47,48^.

A highly organized network of transcriptional factors and tightly regulated epigenetic mechanisms regulate the development of the endocrine pancreas. Therefore, interfering with the NF-κB signaling pathway during fetal life had a selective consequence on the formation of β-cells in postnatal development. Although largely involved in innate and adaptive immunity, NF-κB has also been reported to play an important role in the development of several other types of tissues, including the embryonic limb, liver, bone, lung, and in the peripheral nervous system development^49–54^.

In summary, the present findings suggest a physiological role for the canonical NF-κB pathway in β-cells during the fetal period, but not in adult mice. In concordance with the lack of activated NF-κB in NOD mice, no nuclear translocation of the transcription factor was detected in pancreata from recently diagnosed T1D patients. More in-depth studies are needed to gain a better understanding of physiological regulators of the NF-κB signaling pathway during these stages, as part of an integrated network of signaling events acting in concert to control BCM adaptation to insulin demand.

## Materials/subjects and methods

### Transgenic mice

The ToIβ and the NOD/ToIβ transgenic mouse models were generated and characterized as described^14,55^. They carry the nondegradable IκBα and luciferase genes (∆NIκBα-luciferase), regulated by a tetracycline-responsive element, and the reverse tetracycline transactivator (rtTA) under the control of the rat insulin II promoter (RIP7-rtTA). The NOD/ToIβ mice were monitored for blood glucose levels once a week, between the 8th week and the 35th week of age, using the Accu-Chek performa glucometer (Roche Diagnostics). All animals were maintained in a specific pathogen-free research animal facility. The experiments were conducted in accordance with local ethical guidelines of the Hebrew University Institutional Animal Care and Use Committee.

### Intraperitoneal glucose tolerance test (IPGTT)

The assays were performed as described previously^14^.

### Histology and immunostaining and β-cell mass

Mice were sacrificed and each pancreas was flattened, incubated in 4% paraformaldehyde in ice for 2–4 h, and then incubated in cold 80% ethanol overnight and on the next day embedded in paraffin. Sections of 5 µm were rehydrated, and antigen retrieval was performed using a PickCell pressure cooker in 10 mM citrate buffer pH = 6.0. The following primary antibodies were used: guinea pig anti-insulin (1:200; Abcam), rabbit anti-Ki67 (1:200; Neo Markers), and rabbit anti-p65 (1:50; Neo Markers). Secondary antibodies were from Jackson Immunoresearch Laboratories. DAPI (Thermo Scientific) was used for nuclear counterstain. Apoptosis was assessed using the terminal deoxynucleotidyl transferase (TdT)-mediated dUTP nick end-labeling (TUNEL) assay (Roche, Mannheim, Germany) according to the manufacturer’s instructions: TdT enzyme solution was mixed with fluorescein-dUTP labeling solution (ratio 1:9). The sections were counterstained with anti-mouse insulin antibodies. All immunofluorescence images were captured on an Olympus FlouView FV1000 confocal microscope at ×400 magnification. Cells apoptosis was calculated as a percent of TUNEL-positive nuclei in insulin-positive cells out of the total number of insulin-positive cells per mouse.

All immunofluorescence images were captured on an Olympus FlouView FV1000 confocal microscope. DAB staining was performed using the Histostein-Plus IHC kit, HRP, broad-spectrum (Invitrogen Life Technologies) counterstained with Harris hematoxylin (Leica-microsystems). BCM was calculated as described^23^.

### Human pancreas samples

Participants in the DiViD study were between 25 and 35 years of age and their biopsy specimens were obtained between 3 and 9 weeks after initial diagnosis. The DiViD study was approved by the Norwegian Government’s Regional Ethics Committee and all samples were studied with ethical approval. Control human pancreas: male 59 BMI 24.2 and female 57 BMI 22.5 from Diabetes Research Institute, IRCCS San Raffaele Scientific Institute, Milan, Italy.

### Degree of insulitis

Sections 400 µm apart were stained with hematoxylin/eosin for evaluation. Islet insulitis scores (0–4) were determined as previously described^14^. Results are presented as the percentage of islets/mice in each score category.

### RNA sequencing

RNA was extracted from isolated islets using TRIzol (Invitrogen, Carlsbad, CA, USA). A bulk adaptation of the MARS-Seq protocol^56^ was used to generate RNA-Seq libraries. Briefly, 30 ng of input RNA from each sample was barcoded during reverse transcription and pooled. Following Agencourct Ampure XP beads cleanup (Beckman Coulter), the pooled samples underwent second-strand synthesis and were linearly amplified by T7 in vitro transcription. The resulting RNA was fragmented and converted into a sequencing-ready library by tagging the samples with Illumina sequences during ligation, RT, and PCR. Libraries were quantified by Qubit and TapeStation as well as by qPCR for ActB gene, as previously described^56^. Sequencing was done on a Nextseq 75 cycles high output kit (Illumina). Sequenced reads were aligned to the mm9 genome with Hisat (v.16). Reads were condensed into original molecules by counting the same unique molecular tags (UMI) associated with the same align location. Duplicate reads were filtered if they aligned to the same base and had identical UMIs. Expression levels were calculated and normalized for each sample to the total number of reads using HOMER software (http://homer.salk.edu).

### Real-time PCR

RNA from pools of islets, each pool isolated from 3 weeks-old mice (*n* = 3–4) that were previously treated with Dox, during the embryonic period or from untreated animals, was extracted and converted to cDNA as described^23^. The list of primers is presented in Supplementary Table S2.

### Data presentation and statistical analysis

The data are presented as means ± SEM. Statistical analyses of IPGTT, degree of insulitis, proliferation, apoptosis, and BCM were performed by using the paired Student’s *t* test. Statistical analysis of diabetes incidence was performed using the Kaplan–Meier test and the log-rank test using the MedCalc 12.3.0.0 software. In all tests, *P* < 0.05 was considered statistically significant.

## Supplementary information

S1

S2

S3

Table S1

Table S2
