# Supplementary figures and images for: NF-κB activity during pancreas development regulates adult β-cell mass by modulating neonatal β-cell proliferation and apoptosis
Source: Cell Death Discov. 2021 Jan 4;7:2. doi: 10.1038/s41420-020-00386-9 (PMC7790827; doi:10.1038/s41420-020-00386-9)

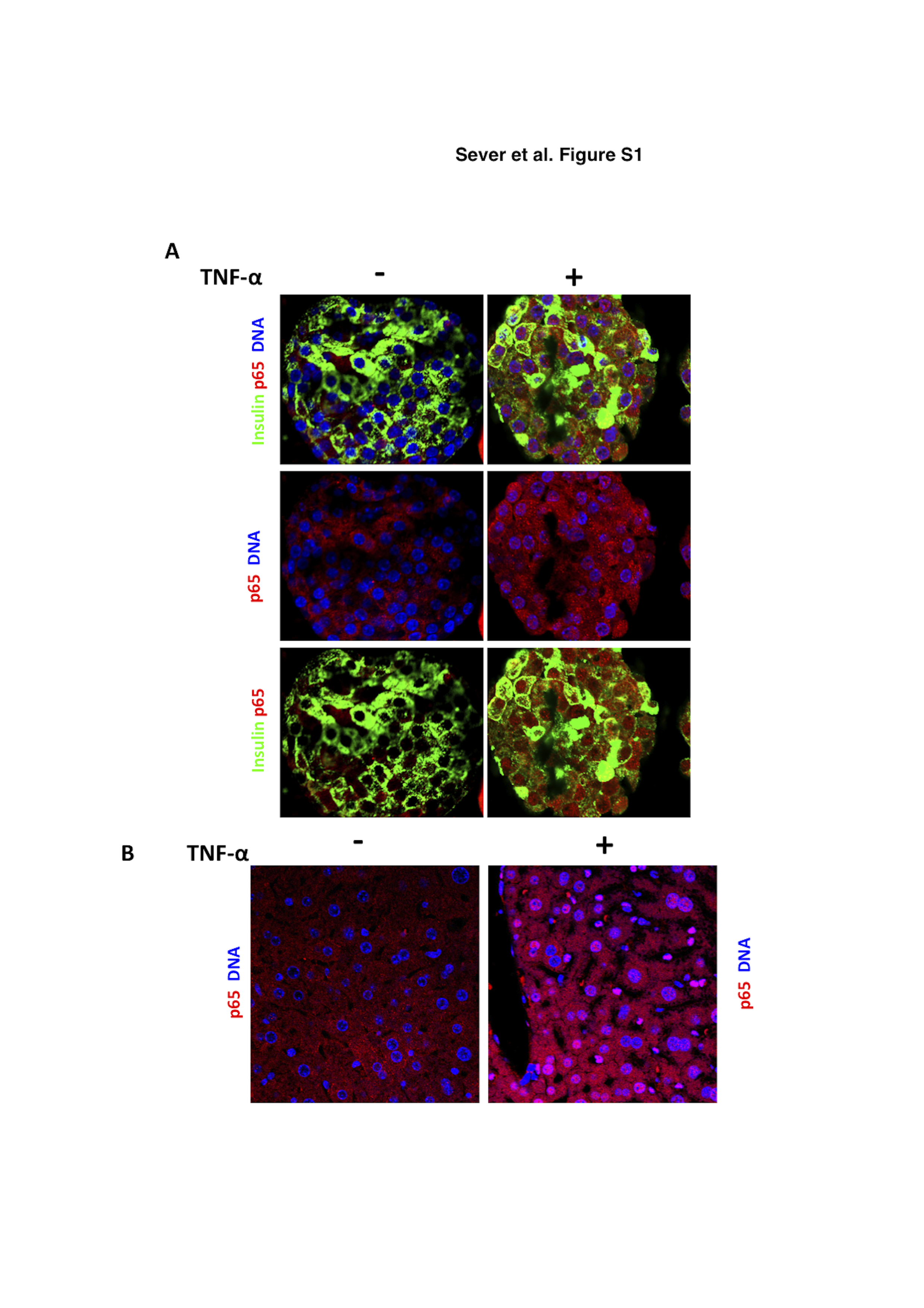

Supplement: Supplementary file 1 — S1 [file 41420_2020_386_MOESM1_ESM.png]

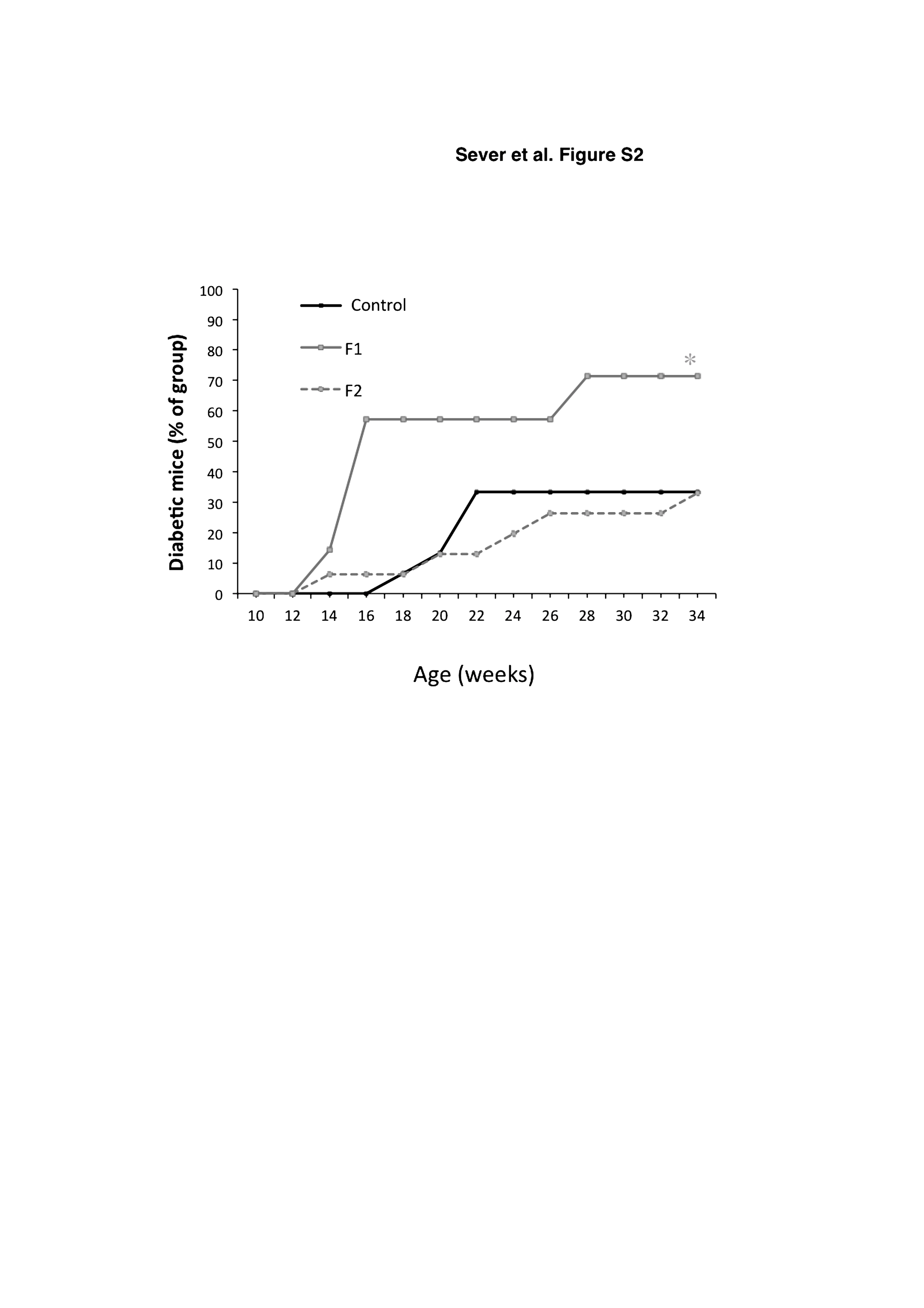

Supplement: Supplementary file 2 — S2 [file 41420_2020_386_MOESM2_ESM.png]

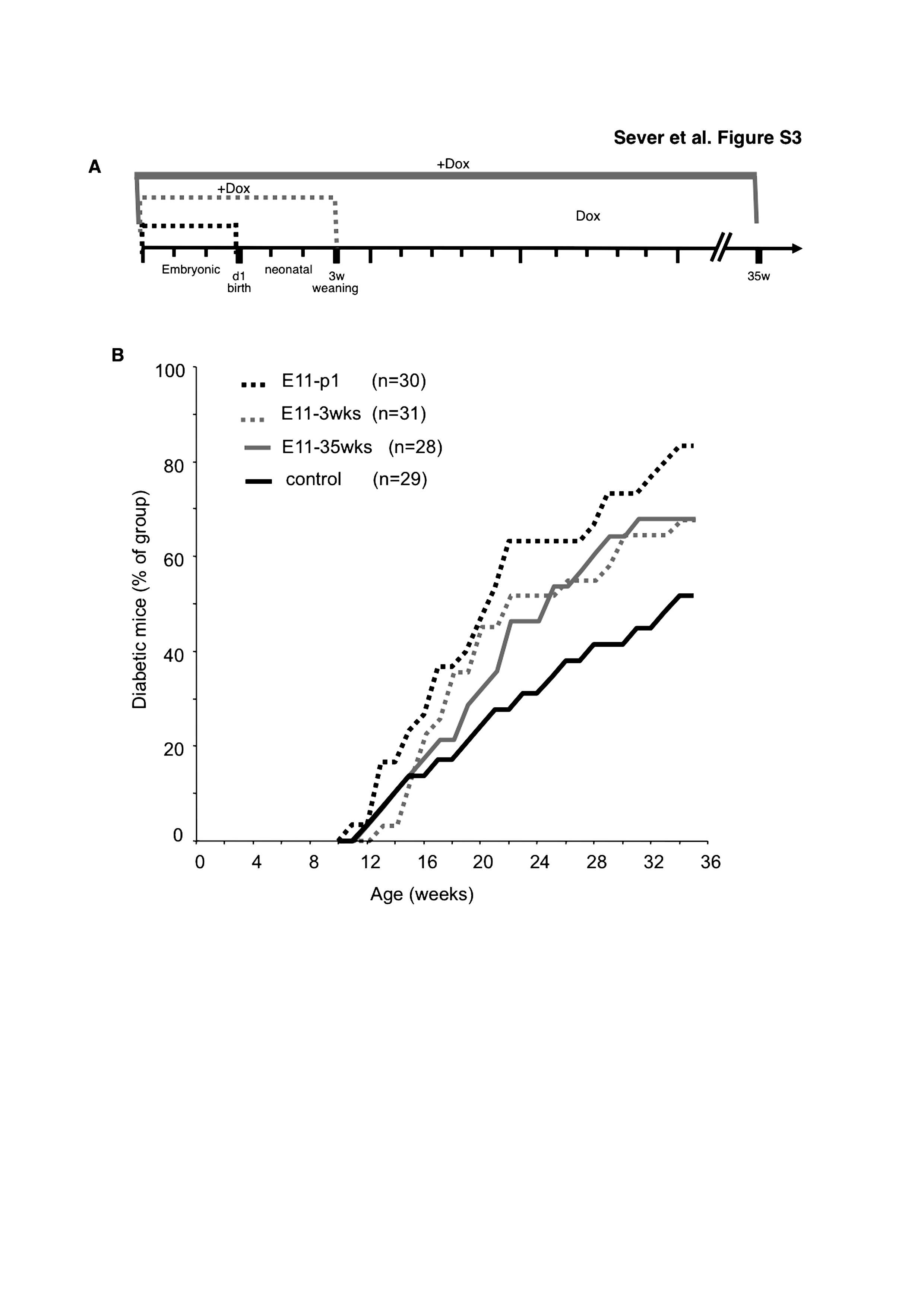

Supplement: Supplementary file 3 — S3 [file 41420_2020_386_MOESM3_ESM.png]
